# Heterotopic ossification in a patient with paroxysmal sympathetic hyperactivity following multiple trauma complicated with vitamin D deficiency: a case report

**DOI:** 10.1186/s40792-020-01031-4

**Published:** 2020-11-23

**Authors:** Takeaki Sato, Mayo Watanabe, Yoshito Onoda, Taku Oyanagi, Shigeki Kushimoto

**Affiliations:** 1 Department of Emergency and Critical Care Medicine, Tohoku University Hospital Emergency Center, 1-1 Seiryo-cho, Aoba-ku, Sendai-shi, 980-8754 Japan; 2 Department of Graduate Medical Education Center, Tohoku University Hospital, 1-1 Seiryo-cho, Aoba-ku, Sendai-shi, Japan; 3 Department of Orthopaedic Surgery, Tohoku University Hospital, 1-1 Seiryo-cho, Aoba-ku, Sendai-shi, Japan

**Keywords:** Heterotopic ossification, Paroxysmal sympathetic hyperactivity, Vitamin D deficiency

## Abstract

**Background:**

Paroxysmal sympathetic hyperactivity (PSH) may occur in patients with traumatic brain injury. Heterotopic ossification (HO) has frequently been observed in patients with PSH and has been found to impair patients’ recoveries. However, the pathophysiology of HO in patients with PSH remains unelucidated. Vitamin D deficiency is a common abnormality among critically ill patients and may be associated not only with musculoskeletal complications, but also with high morbidity and mortality. The association between vitamin D deficiency and HO in patients with PSH has not yet been evaluated.

**Case presentation:**

A 21-year-old man was in a motorcycle accident. The initial diagnosis was diffused axonal injury, thoracic aortic injury, bilateral lung contusion with hemopneumothorax, liver injury, vertebral injury of T5, along with fractures of the right humerus, left patella, bilateral scapula, and a stable pelvic fracture, with an Injury Severity Score of 50. Two weeks after admission, he was diagnosed with PSH. One month after the injury, decreased joint mobility and progressive pain were evident. Computed tomography (CT) showed HO in his humerus, ulna, radius, scapula, ilium, pubis, ischium, knee joint, patella, and tibia, as well as renal calculus. To evaluate metabolic bone abnormalities, we measured levels of 25-OH vitamin D, parathyroid hormone, calcitonin, procollagen type I N-terminal propeptide (a marker of bone formation), and tartrate-resistant acid phosphatase 5b (a marker of bone resorption). This revealed a vitamin D deficiency. Bisphosphonate agents and vitamin D were administered for 1 month. Thereafter, his symptoms, radiographic findings, and laboratory abnormalities improved, and he was transferred to another facility.

**Conclusions:**

HO in patients with PSH, following severe head injury, may be associated with vitamin D deficiency. Medication for vitamin-D-related metabolism abnormalities may represent a novel intervention for HO with PSH.

## Background

In patients with traumatic brain injury, an excessive response of the autonomic nervous system may occur, which is defined as paroxysmal sympathetic hyperactivity (PSH) [1]. Heterotopic ossification (HO) is the presence of lamellar bone in soft tissue, where normally bone does not exist [2]. HO is frequently observed in patients with musculoskeletal trauma, spinal cord injury, and central nervous system injury [3]. HO occurs as a complication in patients with PSH and is considered a diagnostic criterion [4]. However, the pathophysiology of HO in patients with PSH has not been elucidated.

Vitamin D deficiency is commonly seen in critically ill patients, and may be associated with musculoskeletal complications as well as high morbidity and mortality [5]. However, the association between vitamin D deficiency and HO in patients with PSH has not been evaluated.

Here, we report a case of multiple instances of HO in a patient with PSH, following severe head injury and an associated vitamin D deficiency.

## Case presentation

**Fig. 1:**
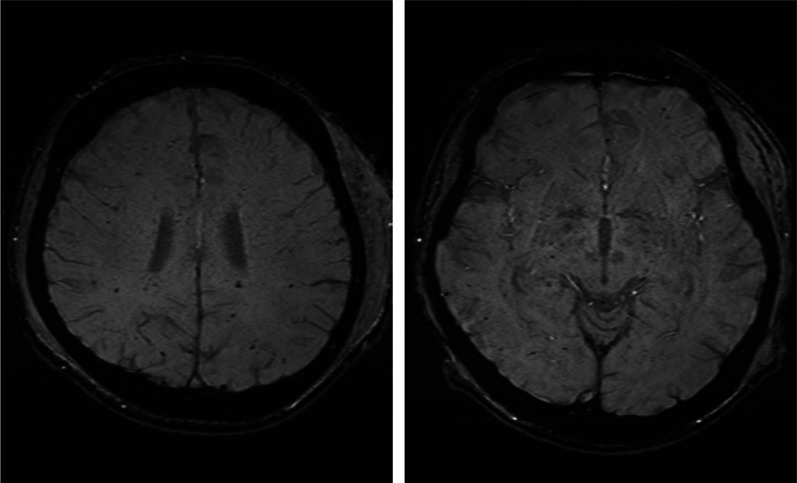
Magnetic resonance images of the brain on the sixth day. Multiple micro-bleeding lesions are observed in the bilateral subcortex, corpus callosum, and bilateral cerebellar hemisphere with susceptibility-weighted images

A 21-year-old male, who was in a motorcycle accident, presented with a confused level of consciousness and had a Glasgow Coma Scale score of E3V4M5. His blood pressure was 58/34 mmHg, his heart rate was 154 beats per minute, and his respiratory rate was 19 breaths per minute on admission. The initial diagnosis was diffused axonal injury, thoracic aortic injury, bilateral lung contusion with hemopneumothorax, liver injury, vertebral injury of T5, along with fractures of the right humerus, left patella, and bilateral scapula, and a stable pelvic fracture, with an injury severity score of 50. Brain magnetic resonance imaging on the 6th day showed diffused microbleeds in the bilateral subcortices, corpus callosum, and bilateral cerebellar hemisphere, consistent with diffuse axonal injury (Fig. 1). A stent-graft repair was performed for the aortic injury, on the same day. On the 10th, 12th, and 17th days, open reduction and internal fixations were performed to repair bone fractures.

After 2 weeks of admission, prolonged drowsiness persisted, and hyperthermia, tachypnea, tachycardia, hypertension, and hyperhidrosis without any specific stimuli were observed paroxysmally several times a day. PSH was diagnosed using the PSH-assessment measure [6].

**Fig. 2:**
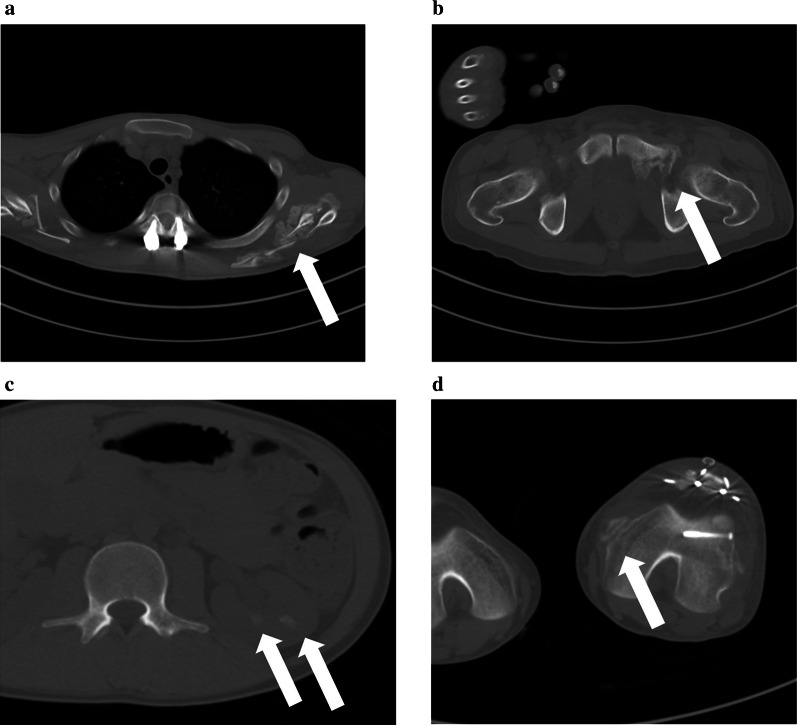
Heterotopic ossifications on computed tomography. Heterotopic ossifications are observed in the scapula (**a**), pubis (**b**), tibia (**c**), and renal calculus (**d**; white arrows)

One month after the injury, decreased joint mobility and progressive pain were evident. Computed tomography (CT) showed HO in his humerus, ulna, radius, scapula, ilium, pubis, ischium, knee joint, patella, and tibia, as well as showing renal calculus (Fig. 2). To evaluate metabolic bone abnormalities, 25-OH vitamin D, parathyroid hormone, calcitonin, N-terminal propeptide of type 1 collagen, and tartrate-resistant acid phosphatase 5b were measured, revealing vitamin D deficiency (Table 1).

**Table 1:** Changes in bone metabolism parameters

	Reference range	1 month	2 months
25-OH-Vitamin D, ng/dL	≥ 20	9.5	17.0
PTH, pg/mL	14.9–56.9	10.7	9.7
Calcitonin, pg/mL	0.5–6.16	< 0.5	< 0.5
P1NP, ng/mL	18.1–74.1	765.1	522.2
TRACP-5b, µg/dL	170–590	1687	457

*PTH* parathyroid hormone, *P1NP* procollagen type I N-terminal propeptide, *TRACP-5b* tartrate-resistant acid phosphatase 5b

A bisphosphonate agent and vitamin D were administered for 1 month to prevent progressive HO and the significant discomfort associated with it. Thereafter, his symptoms, radiographic findings, and laboratory abnormalities improved. On the 55th day, he was transferred to another facility for rehabilitation.

## Discussion

HO reportedly caused by hypercalcemia, tissue hypoxia, changes in sympathetic nerve activity, prolonged immobilization, and disequilibrium between parathyroid hormone and calcitonin [3]. It has also been shown that HO can be induced by fracture, burn, and neurological damage (brain injury and spinal cord injury) [7].

PSH was observed in approximately 10% of patients with severe traumatic brain injury [8]. HO occurred in 13.6% of patients with severe acquired brain injury, and a higher prevalence of HO was observed in male patients, younger patients, and those with PSH [9].

Few studies have sought to evaluate the relationship between vitamin D deficiency and HO in patients with PSH. Although vitamin D deficiency is considered common and is associated with high morbidity and mortality in critically ill patients, there is no evidence that the administration of high-dose vitamin D has any advantage over a placebo with respect to 90-day mortality [10]. However, maintaining therapeutic serum vitamin D levels could reduce the risk of developing HO [11].

In this case, both parathyroid hormone levels and calcitonin secretions were controlled. These hormonal levels would not directly indicate a specific relation between HO and vitamin D deficiency. However, the improvement of HO after the administration of vitamin D and a previous report [11] might support the relation between HO and vitamin D deficiency. Therefore, this finding could contribute to the establishment of vitamin D administration as a therapeutic option in patients at risk of HO.

## Conclusions

HO in patients with PSH following severe head injury may be associated with vitamin D deficiency. Medications for vitamin-D-related metabolic abnormalities could also represent a novel treatment for this complex condition.

## Data Availability

All datasets supporting the conclusions of this article are included within the article.

## Abbreviations

PSH: Paroxysmal sympathetic hyperactivity
HO: Heterotopic ossification
P1NP: N-terminal propeptides of type 1 collagen
TRACP-5b: Tartrate-resistant acid phosphatase 5b
